# How many words make a sample? Determining the minimum number of word tokens needed in connected speech samples for child speech assessment

**DOI:** 10.1080/02699206.2020.1827458

**Published:** 2020-10-06

**Authors:** Yvonne Wren, Jill Titterington, Paul White

**Affiliations:** a Bristol Speech and Language Therapy Research Unit, North Bristol NHS Trust, Bristol, UK; b Bristol Dental School, University of Bristol, Bristol, UK; c Institute of Nursing and Health Research, Ulster University, Newtownabbey, Northern Ireland, UK; d Applied Statistics Group, University of the West of England, Bristol, UK

**Keywords:** Speech, transcription, speech Sound Disorder, alspac, connected speech, sample size

## Abstract

Connected speech (CS) is an important component of child speech assessment in both clinical practice and research. There is debate in the literature regarding what size sample of CS is required to facilitate reliable measures of speech output. The aim of this study was to identify the minimum number of word tokens required to obtain a reliable measure of CS across a range of measures. Participants were 776 5-year-olds from a longitudinal community population cohort study (Avon Longitudinal Study of Parents and Children, ALSPAC). Children’s narratives from a story retell task were audio-recorded and phonetically transcribed. Automatic analysis of the transcribed speech samples was completed using an automated transcription and analysis system. Measures of speech performance extracted included: a range of profiles of percentage consonant correct; frequency of substitutions, omissions, distortions and additions (SODA); percentage of syllable and stress pattern matches; and a measure of whole word complexity (Phonological Mean Length of Utterance, pMLU). Statistical analyses compared these measures at different CS sample sizes in increments using averages and weighted moving averages, and investigated how measures performed between CS samples grouped into word tokens of at least 50, 75 and 100, and restricted to samples of 50–74, 75–99 and 100–125. Key findings showed that sample sizes of 75 word tokens and above showed minimal differences in most measures of speech output, suggesting that the minimum requirement for samples of CS is a word count of 75. The exception to this is in the case of pMLU and measures of substitutions and distortions when a word count of 100 is recommended.

## Introduction

Connected or spontaneous speech samples are collected across a range of play or composite picture activities, and are globally recommended as a fundamental part of the speech and language therapist’s (SLT’s) assessment process for children with speech sound disorders (SSD) (see Bates & Titterington with the Child Speech Disorder Research Network Child Speech Disorder Research Network (2017); Child Speech Disorder Research Network (2017); McLeod & Baker, 2017). In research, connected speech (CS) also often provides essential data from which measures of speech accuracy and phonological ability are gleaned (e.g., Morris, 2009; Smit et al., 2018; Stoel-Gammon, 2010). CS has ecological validity as it reflects functional use of speech in the everyday context better than a single word sample. The complexity required for CS versus single word production also suggests that it may be more sensitive to the everyday impact of SSD. Importantly, it allows the clinician to step back and compare targets produced in single words with those produced in CS and fundamentally, provides additional insight into expressive language ability, suprasegmental skills, speech behaviour across word boundaries and overall intelligibility (Bates & Titterington with the Child Speech Disorder Research Network, 2017; Thompson & Howard, 2007).

### What is the minimum sample length needed to support robust analysis of connected speech?

The reliability and validity of CS performance using single score measures are strongly linked to the size of the CS sample with time efficiency being an important factor for consideration. Clearly, researchers and clinicians alike must counter resource constraints against the amount of data required to provide a sensitive measure. However, advice varies across measures of CS about their optimal sample size and there is little research comparing sample sizes and measures.

Measures used in analysis of CS performance can be summarised as follows:

1. Phonetic/segmental level measures, e.g., Percentage Consonants Correct (PCC) and its extensions consider inventories of correctly realised consonants and/or vowels which may be selected to more sensitively capture SSD and age effects by use of a speech profiling approach, i.e., filtering PCC through the early, middle or late eight acquired phonemes (Shriberg et al., 1997) and the early substitution, omission, distortion and addition (SODA) analysis of Van Riper (1939) which considers frequency of substitutions, omissions, distortions and additions.
2. Syllable and word-level measures consider aspects such as the most frequently used syllable shapes, matching of the production and target, use of complex syllable shapes and stress pattern use (Bernhardt & Stemberger, 2000).
3. Phonological level measures, e.g., phonological processes used, contrastiveness of phoneme use and feature analysis (see Ingram & Ingram, 2001).
4. Whole word level measures score how the entire word is realised considering various phonetic, phonological and complexity factors dependent on the scoring system devised. For example, the Index of Phonetic Complexity (IPC, Jakielski et al., 2006), the Whole Word Complexity Measure (WWC, Stoel-Gammon, 2010) and the Syllable Structure Level (Paul & Jennings, 1992) consider aspects of speech production at word, syllable and segmental levels and are largely independent measures (i.e., the child’s productions are analysed without reference to the adult target) although the WWC is gathered for both the adult target and the child’s productions for comparison purposes. The phonological mean length of utterance (PMLU, Ingram, 2002; Ingram & Ingram, 2001) reflects complexity and degree of segmental accuracy. Scoring involves the child receiving a point for each segment produced and then an additional point for each consonant produced correctly. The sum of these figures is then divided by the total number of words analysed.

Analysis of phonetic inventory to identify the presence/emergence of specific phonemes within a child’s speech sound system is often used within the clinical context (McLeod & Crowe, 2018). However, this qualitative measure is not easily converted to a single score and has also been found to have poor test-retest reliability (Morris, 2009).

As noted in the UK & Ireland’s CSDRN transcription guidelines (Child Speech Disorder Research Network, 2017), general recommendations about the minimum length of a CS sample vary widely, irrespective of the specific measure to be used. There is debate around how to best ensure a CS sample is sensitive to SSD and also representative of a child’s full phonetic inventory and therefore suitable for comparison purposes both for individual children pre, during and post intervention, and between children (McLeod & Baker, 2017). Grunwell (1987) recommended a minimum CS sample size of 100 word tokens when considering children with SSD for any measure of speech used. Shriberg et al. (1997) recommend a five to ten-minute sample (~100 word tokens) for their measure of percentage consonants correct (PCC), increasing to at least 500 words when carrying out research to support classification of SSD. In contrast, specific recommendations about the minimum sample size for SODA analysis when using CS have been lacking. This is despite SODA analysis of CS having added value in identifying the speech characteristics of older children with more persistent SSD (e.g., Wren et al., 2016).

When considering CS measures beyond the segmental level, Jakielski et al. (2006) collected samples that were 60 minutes or 50 utterances long for their investigation of the Index of Phonetic Complexity considering children with typical development. Stoel-Gammon (2010) has argued that her Whole Word Complexity Measure is very similar to the Index of Phonetic Complexity and can provide valuable information even with sample sizes ranging from as low as 35 words for children with and without phonological delay/disorder, although she acknowledges that the optimal sample size has not yet been identified for this measure. When calculating Syllable Structure Level in younger children, Morris (2010) recommends a sample size collating a minimum of 50 single word utterances and vocalisations from her review of studies including both typically developing children and those with language impairment.

Ingram and Ingram (2001) indicate that the sample size for the phonological Mean Length of Utterance (pMLU) measure should be a minimum of 25 but ideally a maximum of 50 words to include nouns, verbs, adjectives and adverbs that are randomly extracted from the speech sample collected (see Ingram (2002) and Ingram and Ingram (2001) for specific guidance). There has been some interesting subsequent research into sample sizes used for pMLU. Watson and Terrell (2012) consideration of 12 typically developing 24–36-month-olds used 50 words extracted from CS samples and showed that pMLU was sensitive to change with age. In Kannada-speakers, Balasubranium et al. (2011) and in Finnish-speakers, Kunnari et al. (2012) respectively found that a minimum of 50 and 100 words extracted from CS were able to differentiate between children with SSD versus those with typical development (despite pMLU being influenced by the ambient language). However overall, participant CS sample sizes across pMLU work have been small and further work is required to investigate the optimal CS sample size for this measure.

Morris (2009) was interested in the temporal stability (immediate test-retest reliability) of several CS measures in toddlers with typical development: phonetic inventory (numbers of initial and final consonants), word shape, syllable structure, and the Index of Complexity. The lack of correlation found between the first and second tests for measures of phonetic inventory in her investigation indicates that the 20-minute CS sample collected was not large enough to result in a reliable measure for these inventory-based measures. In contrast, the whole word measures of Syllable Structure Level (adapted from Paul and Jennings (1992)) and Index of Phonetic Complexity (Jakielski et al., 2006) had a high level of stability between the two test times (although the Syllable Structure Level was the only one to reach a significant correlation). This highlights the potential sensitivity of more complex whole word measures to CS performance even with smaller sample sizes of word tokens in younger children.

It is clear that there is conflicting information regarding the minimum requirements for a connected speech sample which will be used to report measures of speech output. To date, this issue has been considered using data from a range of samples, mostly looking at a restricted range of measures of speech. There has not been a large-scale study using a normative population comparing different speech sample sizes for a range of measures of CS. The purpose of the current study was to consider the question of minimum sample size for a wide range of speech measures using data from a community population study. The work was designed to address the following research question:

What is the minimum number of word tokens required in a sample of connected speech to obtain reliable measures of a child’s speech output?

## Method

This investigation used data from a normative sample of 5-year-old children who were participants in a large-scale prospective longitudinal community population study known as the Avon Longitudinal Study of Parents and Children (ALSPAC). Pregnant women resident in Avon, UK with expected dates of delivery 1 April 1991 to 31 December 1992 were invited to take part in the study. The initial number of pregnancies enrolled was 14,541. Of these initial pregnancies, there was a total of 14,676 foetuses, resulting in 14,062 live births and 13,988 children who were alive at 1 year of age and invited to participate in future data collection.

### Participants

A 10% sample of the ALSPAC cohort, known as the Children in Focus (CiF) group, attended clinics at the University of Bristol at various time intervals between 4 and 61 months of age. The CiF group was chosen at random from the last 6 months of ALSPAC births (1432 families attended at least one clinic). Excluded were those mothers who had moved out of the area or were lost to follow-up, and those partaking in another study of infant development in Avon. Children attending the CiF at 61 months were the participants for this study. Data on the number of children with SSD or typically developing speech were unavailable at the time of analysis however, as this was a normative sample, it would be not unreasonable to assume that the number of children with SSD would be equivalent to that of a typical community population. Invitations were sent to 1432 children and 988 attended. The phases of enrolment are described in more detail in the cohort profile papers (Boyd et al., 2013; Fraser et al., 2013). Please note that the study website contains details of all the data that are available through a fully searchable data dictionary and variable search tool at http://www.bris.ac.uk/alspac/researchers/data-access/data-dictionary/.

### Data collection

Children attending the CiF clinic at age 61 months were assessed on a range of measures of speech and language including single word naming, comprehension and multisyllabic word repetition. They were also assessed on the Bus Story (Renfrew, 1997), a test of narrative ability which generated a connected speech sample. These samples were audio-recorded and used as the basis for this study. All assessments were carried out by qualified speech and language therapists and completed within 20 minutes. More information on the assessment procedure is available in Seifert et al. (2020).

**Figure 1.**
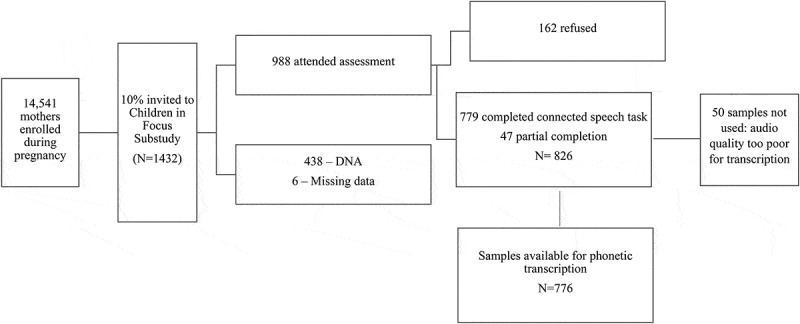
Flowchart showing number of participants at each stage in the study

Of the 988 children who attended the CiF at the 61 months clinic, 779 completed the Bus Story, another 47 partially completed it and 162 did not attempt it. Of the total of 826 recordings, 50 were of poor audio quality and could not be transcribed. The final sample consisted of 776 recordings of connected speech which equates to 78.5% of the sample of children who attended CiF at 61 months (see Figure 1 for flowchart of participant numbers).

#### Phonetic transcription

The recordings were transcribed by a qualified speech and language therapist using the PROPH+ program from Computerized Profiling (Long et al., 2006) which provided an automatic analysis of the speech samples. Given that the recordings were collected from a normative sample, most children exhibited typically developing speech. Broad transcription was used throughout and narrow transcription was used as required for errors in speech production. These errors occurred as a result of typical immature speech, impaired speech production or due to idiosyncratic productions from an otherwise typical speaker.

#### Reliability

A random selection of 10% of the recordings (77) was made for the purposes of inter-rater reliability of transcription. This work was the subject of a separate study investigating different approaches to measurement of transcription reliability. At this time, 14 of the recordings selected were unavailable and therefore repeat transcriptions were carried out on the remaining 63 (8%), also by a qualified speech and language therapist. These paired transcripts were subject to a rigorous reliability check in which matching across the two pairs of samples was carried out separately for different categories of phonemes (Seifert et al., 2020). The overall percentage agreement between the two sets of transcripts was 77% which rose to 82% when those differences which categories as ‘near functional equivalence’ were excluded.

#### Speech analysis

Analysis using the PROPH+ program from Computerized Profiling generated a wide range of measures of speech accuracy. Those used in the statistical analyses were percentage of vowels correct (PVC), percentage of consonants correct (PCC) (Shriberg et al., 1997) and measures of syllable and whole word level accuracy and complexity. Phonetic inventory was not included because of the difficulties in converting to this to a single score.

In addition to the basic PVC and PCC measures, a number of additional measures of subgroups of consonants were also generated and included in the analysis (PCC early 8, PCC middle 8, PCC late 8, PCC-revised (PCC-R), PCC-adjusted (PCC-A) and PCC scores for consonant manner (stops, nasals, fricatives, affricates, glides, liquids) and for clusters, and cluster elements. Measures for percentage of substitutions, omissions (singletons, entire clusters, cluster elements), distortions and additions were also included.

The syllable and whole word level measures used were percentage of word shape matches, percentage of stress pattern matches (Bernhardt & Stemberger, 2000) and phonological mean length of utterance (pMLU) (Ingram & Ingram, 2001). More information on these measures is provided in Wren et al. (2013).

### Statistical analysis

Descriptive statistics were used to provide an overview of the samples in terms of the range of word tokens available across the samples. Results from the PROPH+ analysis using samples with the current recommended minimum of at least 100 word tokens (Grunwell, 1987) were compared with a reduced number of at least 50 word tokens and at least 75 word tokens within speech samples to observe how much change occurred in the measurements of speech. This was carried out to determine whether the current guidance of 100 words for measures of PCC (Shriberg et al., 1997) and up to 50 words for syllable level/whole word measures (Ingram & Ingram, 2001; Morris, 2010) were still appropriate.

A comparison of mean measures of speech accuracy and speech complexity for 50 to 74 word tokens, against 75 to 99 word tokens, and 100 to 125 word tokens was undertaken using a one-way analysis of variance with a post hoc application of Tukey’s HSD to locate any differences between groups. Cohen’s *d* (LeCroy & Krysik, 2007) was used to quantify effect size when comparing the 50 to 74 word token group with the 100 to 125 word token group, and also for comparing the 75 to 99 word token group with the 100 to 125 word token group.

In addition, mean measures of speech accuracy and complexity were calculated for total word token thresholds of 50 through to 125 in increments of 5 (i.e. speech samples with ≥50 word tokens, ≥55 word tokens, through to ≥125 word tokens) and plotted against the number of word tokens, in order to identify any evolving trends with increasing numbers of word tokens. Further, a weighted five-point moving average for each measure of speech accuracy and complexity was calculated over each moving window spanning consecutive ranges of total number of word tokens, from windows, 48 word tokens to 52 word tokens (midpoint 50), 49 to 53 (midpoint 51), 50 to 54 (midpoint 52), and so on through to the final window of 123 word tokens to 127 word tokens (midpoint 125). Each five-point weighted average of speech accuracy was plotted against the window midpoint, so as to graphically identify any trends in localised mean value with increasing number of word tokens.

## Results

Across all 776 samples of connected speech, the number of word tokens ranged from 1 to 175, with a mean of 83 and a standard deviation of 39. The means and standard deviations for all of the measures generated by PROPH+ were calculated for samples of at least 50 word tokens (n = 608), of at least 75 word tokens (n = 491), and of at least 100 word tokens (n = 293) as shown in Table 1. The data in Table 1 comprise overlapping groups (e.g., out of necessity, those with ≥100 word tokens will be in the ≥50 word token group, and in the ≥75 word token group) and as such are given descriptively. In contrast, Table 2 summarises means and standard deviations for those with between 50 and 74 word tokens inclusive (n = 117), with between 75 and 99 word tokens inclusive (n = 198), and those with between 100 and 125 word tokens inclusive (n = 186). These non-overlapping groups are described statistically.

**Table 1.** Percentage correct for Vowels, Consonants (various), Consonant Manner (Stops, Nasals, Fractives, Affricates, Glides, Liquids), Clusters, Cluster Elements, Omissions, Additions, Distortions WSM, SPM, and pMLU

	≥ 50 Tokens		≥ 75 Tokens		≥ 100 Tokens	
	Mean	SD	Mean	SD	Mean	SD
PVC	97.96	2.219	98.19	1.839	98.39	1.636
PCC	89.57	5.977	89.86	5.750	90.18	5.492
PCC Early 8	94.30	5.090	94.44	5.076	94.53	5.081
PCC Middle 8	90.02	8.280	90.42	7.514	90.91	6.866
PCC Late 8	80.68	11.259	81.13	10.704	81.71	10.076
PCC-Revised	94.61	3.657	94.88	3.324	95.15	3.116
PCC-Adjusted	90.72	5.407	91.00	5.189	91.36	4.978
PCC Stops	92.80	7.962	93.32	6.360	93.50	6.168
PCC Nasals	95.30	8.139	95.44	8.247	95.38	8.535
PCC Fricatives	84.51	10.980	85.03	10.129	86.24	8.763
PCC Affricates	78.66	34.722	80.67	32.596	79.87	32.758
PCC Glides	94.09	10.475	94.44	9.552	94.64	8.634
PCC Liquids	84.22	14.856	84.56	14.194	84.28	14.240
PCC Clusters	81.27	12.402	81.42	12.020	81.46	11.640
PCC Cluster Elements	87.81	8.707	87.89	8.548	88.01	8.198
Substitutions	25.28	19.436	24.32	18.991	22.72	18.210
Omissions singletons	2.47	6.143	2.43	6.143	2.42	5.977
Omissions entire cluster	0.08	0.932	0.10	1.030	0.08	1.082
Omissions cluster element	3.18	6.128	3.18	5.935	2.93	4.888
Distortions	50.66	21.140	51.75	20.353	53.47	19.248
Additions	17.65	13.267	17.83	13.303	18.05	12.652
Word Shape Matches	95.18	2.676	95.23	2.912	95.43	2.674
Stress Pattern Match	98.78	4.121	99.04	1.159	99.15	1.048
pMLU	4.954	0.3391	4.992	0.3242	5.038	0.2966

**Table 2.** Mean and standard deviation for the group providing between 50 and 74 word tokens inclusive, between 75 and 99 word tokens inclusive, and between 100 and 125 word tokens inclusive, *p*-value from an analysis of variance for a one-way between subjects design. d_1_and d_2_ is Cohen’s *d* for comparing the 50 to 74 group, and the 75 to 99 group with the 100 to 125 group respectively

	50 to 74 Tokens		75 to 99 Tokens		100 to 125 Tokens		p-value	$d_{1}$	$d_{2}$
	Mean	SD	Mean	SD	Mean	SD			
PVC	96.95	3.232	97.91	2.073	98.49	1.549	**<.001**	0.16	0.08
PCC	88.24	6.755	89.42	6.073	89.86	5.752	.081	0.06	0.02
PCC Early 8	93.69	5.092	94.32	5.091	94.44	5.378	.454	0.04	0.01
PCC Middle 8	88.19	10.860	89.74	8.323	90.23	7.426	.131	0.06	0.02
PCC Late 8	78.75	13.215	80.29	11.555	81.26	10.399	.189	0.05	0.02
PCC-Revised	93.40	4.668	94.46	3.585	94.93	3.297	.**003**	0.10	0.03
PCC-Adjusted	89.46	6.147	90.49	5.436	91.02	5.251	.062	0.07	0.02
PCC Stops	91.31	9.232	92.64	9.324	93.10	6.583	.191	0.06	0.01
PCC Nasals	94.66	7.552	95.53	7.845	95.19	9.410	.683	0.02	0.01
PCC Fricatives	82.20	13.799	83.22	11.703	85.92	9.377	.**011**	0.08	0.06
PCC Affricates	68.64	42.196	82.00	32.457	78.33	33.100	.**012**	0.06	0.03
PCC Glides	92.61	13.681	94.17	10.807	94.55	9.152	.312	0.04	0.01
PCC Liquids	82.93	17.362	84.96	14.158	83.68	14.430	.479	0.01	0.02
PCC Clusters	80.34	14.696	81.62	12.080	80.78	11.869	.655	0.01	0.02
PCC Cluster Elements	87.36	9.670	87.83	8.884	87.65	8.267	.904	0.01	0.01
Substitutions	29.21	20.787	26.84	19.943	23.46	18.247	.**038**	0.07	0.04
Omissions singletons	2.68	6.141	2.45	6.425	1.93	5.087	.514	0.03	0.02
Omissions entire cluster	.000	.0000	0.11	0.9536	.09	1.224	.598	0.04	0.00
Omissions cluster element	3.32	6.880	3.55	7.229	3.16	5.095	.840	0.01	0.02
Distortions	46.07	23.811	49.05	21.742	52.72	18.933	.**027**	0.08	0.05
Additions	16.98	12.934	17.50	14.310	18.65	13.130	.538	0.03	0.02
Word Shape Matches	94.93	3.229	94.93	3.233	95.41	2.723	.235	0.04	0.04
Stress Pattern Matches	98.48	1.871	98.39	6.942	99.14	1.026	.244	0.11	0.05
pMLU	4.790	0.3545	4.923	0.3512	5.023	0.2902	**<.001**	0.18	0.08

Based on Table 1, the absolute difference in means between samples with a threshold of 50 word token, with a threshold of 75 word tokens and a threshold of 100 word tokens are arguably small and minor and the ratio of standard deviations do not majorly differ from a ratio of 1. However, this may be expected as a data artefact as those meeting the criteria of word token threshold ≥100, by definition, also meet the criteria of word token threshold ≥75.

Table 2, comparing samples with between 50 and 74 word tokens, 75 to 99 word tokens, and 100 to 125 word tokens, similarly suggest the characteristics of these three groups are similar.

In Table 2, there are statistically significant differences between groups on PVC, PCC-Revised, Fricatives, Affricates, Substitutions, Distortions and pMLU. Tukey’s HSD test was used to examine all pairwise group differences. For all measures, where the ANOVA *p*-value was >0.05 the three groups (50 to 74, 75 to 99, and 100 to 125 word tokens) did not show any differences in mean values. The ANOVA for PVC, PCC-Revised, PCC-Fricatives, PCC-Affricates, and Substitutions, all show significant effects, but in each and every case there is no significant difference in mean values between the 75 to 99 group and the 100 to 125 group.

In all cases, Cohen’s *d* indicates that the effect is largest when comparing the 50 to 74 group with the 100 to 125 group (i.e. $d_{1}$ < $d_{2}$) and the effect when comparing the 75 to 99 group with the 100 to 125 group is not always in the same direction (mean difference sometimes positive and other times negative). In all cases the degree of systematic bias between the 75 to 99 group with the 100 to 125 group has an absolute value of $d_{2}$ ≤ 0.08.

**Figure 2.**
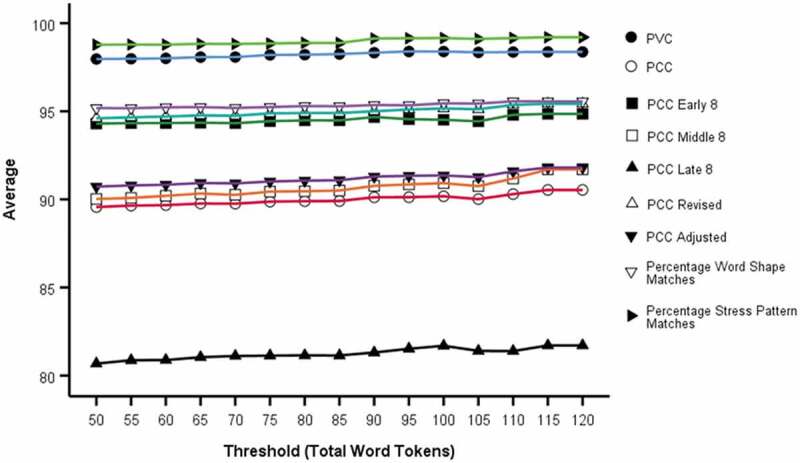
Average (mean) value for Word Token thresholds (PVC, PCC-various, syllable measures) from 50 to 125 in increments of 5

Inspection of Figure 2 broadly indicates mean values for PVC, PCC-Revised, PCC Early 8 and word shape matches, stress pattern matches are largely invariant to chosen threshold with no discernible trend with word token threshold. There is arguably a small positive trend for PCC, PCC middle 8, PCC late 8 and PCC-Adjusted with increasing threshold.

**Figure 3.**
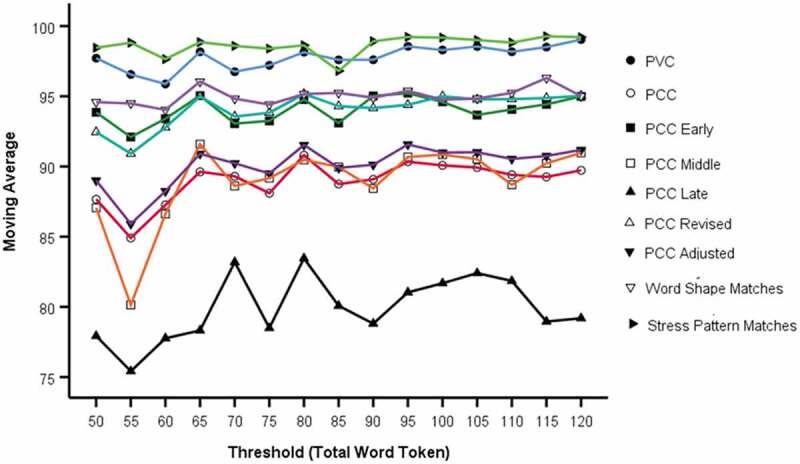
Five-point moving average for Word Token thresholds (PVC, PCC-various, syllable measures)

Out of necessity, there is an increased variation in Figure 3 compared with Figure 2 due to each five-point window in Figure 3 comprising a smaller number of data points compared to using a threshold which includes all data points meeting that threshold. Inspection of Figure 3 suggests that localised mean levels on all measures have no discernible trend beyond 75-word tokens.

**Figure 4.**
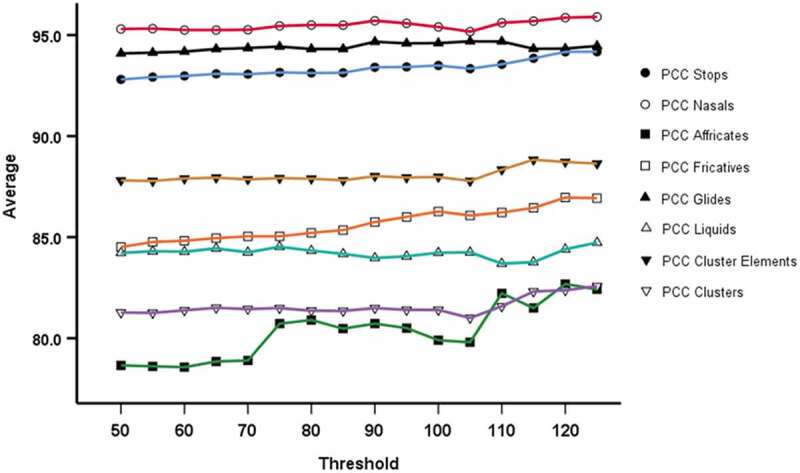
Average (mean) value for Word Token thresholds (PCC for manner and for clusters) from 50 to 125 in increments of 5

Figure 4 shows that mean PCC Affricates show a marked step jump at 75-word tokens, and PCC Stops, Affricates, Fricatives and Clusters tend to show an increasing trend in mean values with increasing word token threshold.

**Figure 5.**
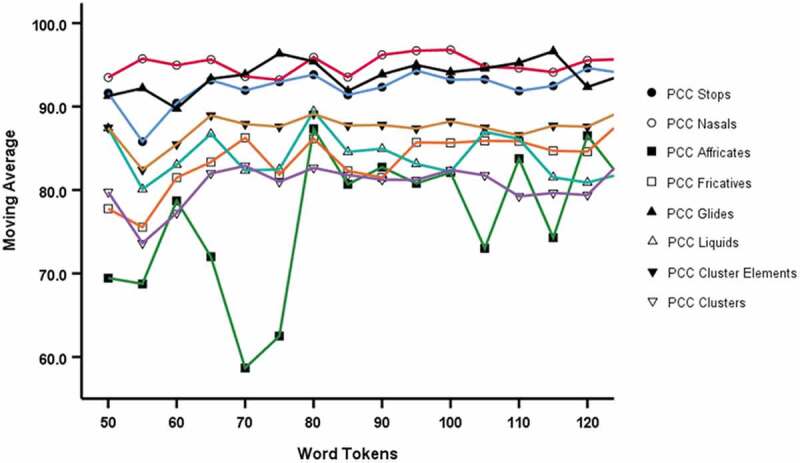
Five point moving average for Word Token thresholds (PCC for manner and for clusters)

In Figure 5, the localised mean characterised by the five-point weighted moving average, shows a marked jump at 75 word tokens for PCC Affricates but which otherwise shows stability in mean values from 80 word tokens onwards. No obvious trend was observed for the other measures.

**Figure 6.**
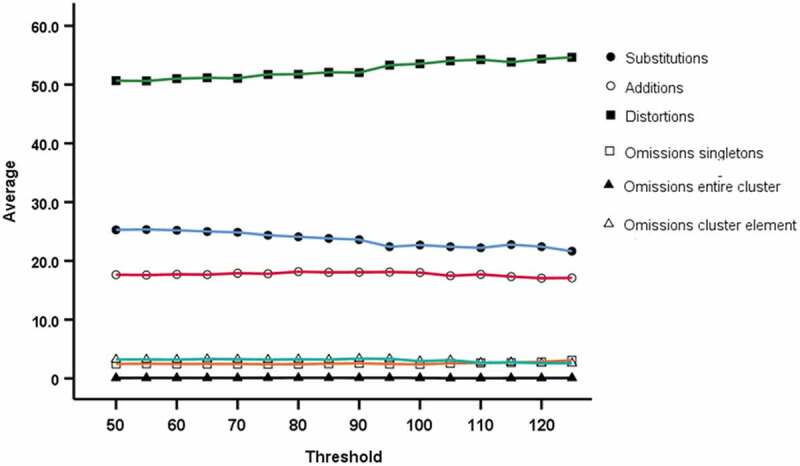
Average (mean) value for Word Token thresholds for percentage substitutions, omissions (singletons and clusters), distortions and additions from 50 to 125 in increments of 5

**Figure 7.**
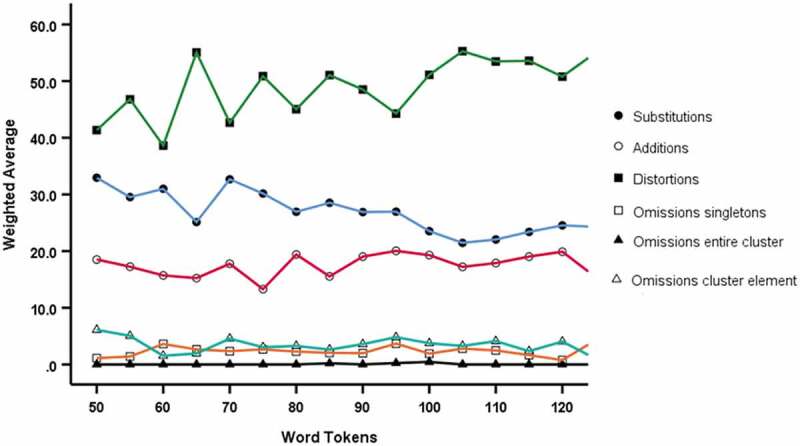
Moving Average Chart for Word Token thresholds for percentage substitutions, omissions (singletons and clusters), distortions and additions

Based on Figures 6 and 7, the suggestion is that percentage substitutions are overestimated at lower word token thresholds compared to higher thresholds (negative trend), whereas percentage distortions are underestimated at lower thresholds compare to higher thresholds (positive trend), with similar trends in the localised mean (five-point moving average). Analysis using Spearman’s rank correlation indicates that both of these trends are statistically significant (*p* <.001). Accordingly, although mean values do not differ between the 75– 99 group and the 100– 125 group, there is a small trend in some of the SODA measures with number of word tokens and a cautionary approach would favour the use of the higher 100 token threshold.

**Figure 8.**
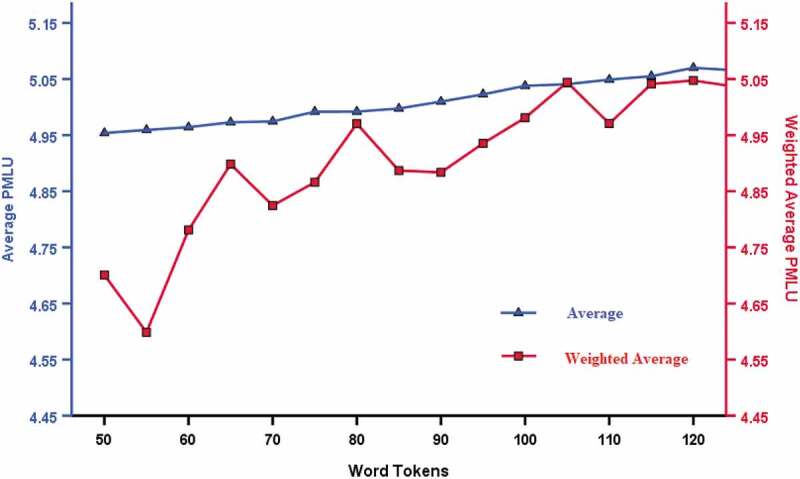
Average (mean) value and moving average chart for pMLU from 50 to 125 in increments of 5

Figure 8 shows both average (mean) values and the moving average values for pMLU. Unlike the other measures of speech included in these analyses, pMLU does not provide a percentage but provides a score which reflects the length of the word produced and the accuracy of its production. The total score for a sample of connected speech is divided by the number of utterances produced in the sample. Scores are expected to rise with increasing complexity of speech. The results in Figure 8 show a rise in value of pMLU as sample size increases regardless of whether average (mean) value or the weighted average is used. The weighted average scores suggest greater variability in pMLU in samples with fewer word tokens though the gap narrows between the localised mean and the mean based on the threshold as sample size increases. Accordingly, although mean values do not differ between the 75– 99 group and the 100– 125 group, there is a non-ignorable trend in pMLU with number of word tokens, to such an extent as to warrant using this measure with at least 100 word tokens.

## Discussion

The aim of this study was to determine how many word tokens are necessary to provide a reliable child speech sample for use in clinical work and research. Previous research has suggested a range based on the length of the sample in time and the length in number of utterances and word tokens but most of these were based on small data sets or considered a single measure or small number of measures of speech accuracy or complexity.

A large community population study in the UK had collected recordings of children’s connected speech at age 5 from over 700 children which were available and could be used to address the study question. These samples were transcribed and analysed using a computerised tool to provide consistent and reliable measures of speech including a range of: percentage phonemes correct measures; percentage of substitutions, omissions, distortions and additions measures; two measures of syllable structure; and one whole word level measure. Data from these measures were used in statistical analysis to compare scores at different sample sizes based on number of word tokens. Mean measures of accuracy and complexity of speech were also calculated for total word token thresholds in increments of five, supplemented by an analysis using a weighted five-point moving average, to identify any trends which could be observed across increasing sample sizes.

The results of these analyses suggested that samples of 75 word tokens showed little difference from that of samples containing 100 words, whether that was inclusive of all samples containing up to 75 and 100 words or samples which contained between 51 and 75 words and between 76 and 100 words. Although some individual measures showed statistically significant differences between the two cut offs (specifically, PVC, PCC-R, PCC Fricatives, PCC-Affricates, percentage substitutions, percentage distortions and pMLU), the analyses suggest that the difference in the effect size for this difference is small.

In the one-way analysis of variance the measures did not show statistically significant differences between the three cut-offs (50– 74, 75– 99, 100– 125) apart from PVC, PCC-R, PCC Fricatives, PCC-Affricates, percentage substitutions, percentage distortions and pMLU. On these measures, the differences between the 75– 99 group and the 100– 125 group were not significantly different and the non-significant estimated effect size was very small. The 50– 74 group did show significant differences between the other two groups.

These findings indicate that as the number of word tokens increases, measures of speech accuracy and complexity also increase and there is less variation in accuracy. The small difference between the two larger samples indicates that a threshold of 75 word tokens or larger would have merit.

Analyses across sample sizes at five-point intervals using the average (mean) value and the weighted moving average showed some trends towards increasing score with increasing sample size. Some of these were slight (PCC, PCC Middle 8, PCC Late 8, PCC-A), others were more obvious (PCC Stops, Affricates, Fricatives and Clusters, percentage distortions, percentage substitutions, pMLU). The lower numbers of word tokens overestimated the percentage of substitutions and underestimated the percentage of distortions compared with larger sample sizes and it may be cautionary to place reliance on the higher 100 word token threshold for the SODA measures. In the case of other measures, no discernible trends were observed with the exception of PCC Affricates and pMLU. PCC affricates showed a marked increase at 75 words in both the average (mean) value and the weighted moving average analyses. This increase for PCC Affricates may be a chance idiosyncratic sample artefact as scores stabilise at 80 word tokens. When considering pMLU, the weighted moving average clearly showed greater variability of score with lower samples of word tokens, stabilising to a greater extent from 75 word tokens onwards but with a non-ignorable statistically significant trend.

In summary, the findings from this study suggest that single score measures of 75 word tokens from a CS sample are as sensitive to the speech skills of a normative sample of 5-year-olds as 100 word tokens for most measures. However, the stability of these CS measures is lost once the sample size drops to 50 word tokens. The evidence suggests that there are, however, non-ignorable trends for pMLU, percentage substitutions and percentage distortions and therefore it would be appropriate to use a higher threshold for these measures. This is valuable new information for clinicians and researchers alike, providing novel information on the stability of several single score measures for different CS sample sizes across the same participants.

### What does this mean for clinicians and researchers?

The findings from the current study suggest that a minimum sample size of 75 word tokens is sufficient to use in analysis to provide robust measures of speech accuracy and complexity in children aged 5, although a higher threshold may be sensible for pMLU and measures of substitutions and distortions. Previous guidance regarding the recommended sample size for CS to use in calculations for speech accuracy using PCC has indicated that larger numbers of word tokens are necessary. Shriberg et al. (1997) gathered samples of 80–270 word tokens (mean: 196.2; standard deviation: 42.7) and generally recommend using a sample of ~100 words progressing up to ~500 word tokens when considering research into classification of SSD. It is possible that the lack of sensitivity to phonological complexity in PCC measures often commented on by researchers (e.g., Rvachew et al., 2004; Smit et al., 2018) may be improved to some extent by the use of a larger number of word tokens in the CS sample. However, findings from the current study suggest that this type of measure stabilizes at ~75 word tokens for most measures and such findings could have an impact on efficiency of data collection for researchers and clinicians alike.

When considering SODA measures, omissions and additions were similar and predictable across sample sizes. However, a significant decrease in substitutions and conversely, a significant increase in distortions was found as the number of word tokens increased. It would be reasonable to assume that to a large extent, those children who produced larger CS samples did so because they had more proficient expressive language and speech skills than those who produced smaller samples for the same task used in this study. Indeed, Morris (2010) highlights that size of vocabulary is linked to both segmental and syllable level skills of speech production. Substitutions usually capture natural phonological processes which typically developing children suppress as their phonology matures, e.g.,, fronting of /t/to [k] as in [ti] for/ki/(‘tea’ for ‘key’). Consequently, it is possible that the finding of fewer substitutions with increased sample size reflects the performance of children with more proficient expressive language and mature phonological systems than those in the group who produced smaller CS samples. A larger sample size may also have impacted on the incidence of distortions which are specifically associated with motor/articulatory errors (in contrast to the phonological errors potentially captured through substitutions). As the speech and language ability of pre-schoolers increases, Watson and Terrell (2012) observe that they naturally attempt to use more complex, polysyllabic words. This, alongside the use of longer, more complex utterances place skills of articulatory sequencing under pressure, potentially triggering a rise in motor/articulatory errors at this young age. Whatever the potential explanations for these findings, it is clear that more research is required investigating sample sizes, complexity of word use, and level of child speech and language ability to fully understand how sample size interacts with SODA.

With regards to measures of syllable structure such as word shape matching and stress pattern matching, work by Morris (2010) using the Syllable Structure Level indicated that a minimum of 50 vocalisations should be gathered to inform its calculation for under 3 s with speech and language difficulties. Similarly, Ingram and Ingram (2001) suggested 25–50 different word tokens should be extracted from a CS sample to reliably calculate pMLU, a measure of speech complexity. Findings from the current study indicate that irrespective of the size of the overall CS sample, the extracted number of word tokens for analysis will be unstable if it is below 75 word tokens for most measures and below 100 word tokens for pMLU and measures of substitutions and distortions.

The reason for such discrepancies between our findings and those of others could be related to the size of the population sampled in previous investigations. For example, Watson and Terrell (2012) and Barasubranium et al. (2011) found that a sample size of 50 word tokens to calculate pMLU was sensitive to differences in age, language and speech skills for children ranging from 2- to 6-years-of-age but the populations sampled were small, ranging from 12 to 46 individuals. It is also possible that phonological measures like pMLU and syllable measures are more sensitive to differences in speech production with smaller CS samples because they capture complexity more readily than phonetic level measures; and that this is particularly influenced by age and presence of SSD.

### Limitations

While this investigation has provided detailed information regarding the perceived added value of speech samples of increasing length in terms of number of word tokens, there are some limitations in the work which should be borne in mind. In particular, the benefits of using data from a large community population sample are balanced with the challenges this creates.

The collection of speech samples had taken place following a given protocol to ensure that all data collection was completed within a given time limit. An alternative approach would have been to collect connected speech samples using a protocol which allowed the facilitator to probe and encourage the child to produce the best and longest sample they were capable of. This would have facilitated an analysis of the differences in time taken to collect samples of different word lengths as well as an analysis of the benefits of longer samples based on word token cut-offs within speech samples.

The samples used in the analyses have differed at each of the cut-off boundaries. As stated before, there is an overlap between the samples in that those in the group of children with more than 100 word tokens will also be in the group of children with more than 75 word tokens. However, the reverse is not true. An alternative approach would be to limit the analyses to only those children with the maximum of 125 word tokens and compare results at each of the cut-off points. This would result in a much smaller sample size, potentially limiting the possible statistical analyses. This would however be useful work for future studies to consider.

It is also important to remember that these analyses have been carried out using data from a normative population. It is possible that a clinical population of children with SSD might show different results, suggesting the need for longer samples or – potentially-shorter samples. Such an analysis could be carried out in the future where large samples of children with SSD are available. In the meantime, the findings reported in this study provide confidence for clinicians who are seeking to determine what sample size is sufficient to distinguish children with SSD from those with typically developing speech.

## Conclusions

The findings of this study suggest that a sample size of 75 word tokens may be sufficient for a robust, sensitive and replicable measure of CS in a normative population of 5-year-olds, with a recommended increased threshold of 100 word tokens for pMLU and measures of substitutions and distortions. Consequently, reliability may not be impacted on by reducing the number of word tokens to 75 for phonetic level measures and may be improved by increasing the number of word tokens to 75 for word-level measures. This evidence supports the collection of speech samples with a minimum of 75 word tokens to use with one or more of the measures included in this study to collect baseline and outcome data in clinical practice and to carry out a range of investigations in research, thereby increasing efficiency of practice.
